# *NOS1* hypermethylation may participate in the colorectal cancer development and be associated with its prognosis

**DOI:** 10.1080/15592294.2026.2660022

**Published:** 2026-04-28

**Authors:** Zhaohui Zhang, Aibuta Yeerken, Xinyao Shao, Sangni Qian, Shujuan Lin, Simeng Gu, Xiaojiang Ying, Zhenjun Li, Xinglin Fei, Jinhua Yang, Mengling Tang, Jianbing Wang, Mingjuan Jin, Kun Chen

**Affiliations:** a Department of Public Health, Second Affiliated Hospital, Zhejiang University School of Medicine, Hangzhou, China; b Department of Environmental Health, Zhejiang Provincial Center for Disease Control and Prevention, Hangzhou, China; c Department of Anorectal Surgery, Shaoxing People’s Hospital, Shaoxing, China; d Jiashan Institute of Cancer Prevention and Treatment, Jiaxing, China; e Department of Public Health, Fourth Affiliated Hospital, Zhejiang University School of Medicine, Hangzhou, China; f Department of Public Health, National Clinical Research Center for Child Health of the Children’s Hospital, Zhejiang University School of Medicine, Hangzhou, China

**Keywords:** Colorectal cancer, colorectal adenoma, DNA methylation, NOS1, carcinogenesis

## Abstract

Colorectal cancer (CRC) is a leading cause of cancer-related deaths worldwide. Although epigenetic alterations are common in CRC, the epigenetic changes that occur during colorectal carcinogenesis remain unclear. Thus, we sought to elucidate the role of *NOS1* methylation in colorectal carcinogenesis, which is essential for understanding disease pathology. We used the UCSC Xena database to comparatively analyze *NOS1* expression and methylation status between tumour and adjacent normal tissues across 33 cancer types. Low expression and hypermethylation of *NOS1* have been identified in 12 cancer types, with CRC demonstrating this characteristic epigenetic regulation. *NOS1* promoter methylation status was examined using genomic DNA extraction and MassARRAY EpiTYPER methylation analysis. *NOS1* hypermethylation was confirmed among CRCs in in-house dataset 1 (*p* < 0.001), and among CRCs and advanced adenomas in in-house dataset 2 (*p* < 0.001). An upward trend in methylation changes was identified from non-advanced adenoma to advanced adenoma to CRC (*p* for trend < 0.001). Quantitative real-time PCR was used to analyze *NOS1* expression in in-house Dataset 3, and significantly low *NOS1* expression was also identified in CRCs (*p* < 0.001). Kaplan-Meier estimator and Cox proportional hazard models were used to assess the prognostic and predictive roles of *NOS1*. *NOS1* hypermethylation was significantly associated with better disease-specific survival (DSS) in CRC patients. *NOS1* hypermethylation is an epigenetic driver of colorectal tumorigenesis and is associated with better survival.

## Introduction

Colorectal cancer (CRC) is a critical public health problem, as it is the third most commonly diagnosed cancer and second leading cause of cancer-related deaths worldwide [1]. The classical pathway of colorectal tumorigenesis is characterized by an adenoma-carcinoma sequence that may last for decades and requires the progressive accumulation of genetic and epigenetic alterations [2,3]. As malignant transformation unfolds gradually, the promising prognosis of CRC hinges on early detection and timely intervention [4]. However, despite advancements, the challenges in cancer screening and surveillance persist owing to the limitations of existing biomarkers. This highlights the imperative to broadly explore additional factors involved in CRC development, specifically investigating the epigenetic regulation of genes that have not yet been associated with tumorigenesis. Such an exploration contributes to a more holistic understanding, bringing us closer to accurately distinguishing adenomas with malignant potential.

Epigenetic changes are closely associated with tumorigenesis, and epigenetic mechanisms such as DNA methylation have been revealed to play a critical role in the initiation and progression of cancer [3–5]. Aberrant methylation, characterized by promoter hypermethylation and transcriptional silencing of tumour suppressor genes such as *CDKN2A*, *MLH1*, and *APC*, is a hallmark event at the early stages of colorectal carcinogenesis [6–8]. In addition, differences in methylation profiles have been shown to affect the likelihood of polyps progressing to CRC. DNA methylation patterns with significant differences have been proposed as biomarkers for cancer diagnosis, prognosis, and prediction of treatment response [9,10]. SEPT9 and the combination of NDRG4 and BMP3 are two methylation-based diagnostic biomarkers approved for CRC, and SDC2, VIM, APC, MGMT, SFRP1, SFRP2, and NDRG4 have distinct functions linked to colorectal tumour progression [11]. Given the complexity of CRC development and the potential involvement of various factors, adopting an initially broader analysis across diverse cancers is a strategic approach to unveiling novel potential biomarkers for CRC. Although cancers possess their own genetic identities, epigenetic modifications are characteristic of all cancers.

*NOS1*, located on chromosome 12q24.2, produces several alternatively spliced mRNA transcripts that can result in five diverse protein isoforms [12–14]. The NOS1 protein, also known as neuronal Nitric Oxide Synthase (nNOS), is an important enzyme predominantly present in neurons and endothelial cells, and is responsible for the generation of nitric oxide. The neuron-specific functions of NOS1 have been well documented [15,16]. However, the role of NOS1 in non-neuronal tissues has received little attention. It is worth noting that previous studies have reported the involvement of NOS1 in the progression of cardiovascular disease, obesity, and various cancers, including colon cancer, nasopharyngeal carcinoma, and melanoma [17–19]. Moreover, studies have identified *NOS1* as a susceptibility gene or prognosis-related gene in CRC [20–22], presenting additional evidence for the involvement of *NOS1* in colorectal tumorigenesis and metastasis. However, the role of *NOS1* methylation in CRC development remains unclear.

In this study, we systematically evaluated the expression and methylation status of *NOS1* in various cancers, using data from the UCSC Xena database. We confirmed *NOS1* hypermethylation in in-house dataset 1 among 48 CRC patients and further validated it using in-house dataset 2, which included an expanded sample size comprising 286 CRC patients, 81 advanced adenoma (AA) patients, and 81 non-advanced adenoma (NAA) patients. Furthermore, the expression levels of *NOS1* in lesion and paired normal tissue samples were determined in in-house dataset 3, which included 68 CRCs, 45 AAs, and 40 NAAs, using quantitative reverse transcription-polymerase chain reaction (qRT-PCR). Moreover, Gene Set Enrichment Analysis (GSEA) using RNA count data of CRC patients from the UCSC Xena database was performed. Finally, we investigated the impact of *NOS1* on disease-specific survival (DSS) in patients with CRC based on data from the UCSC Xena database and in-house datasets.

## Materials & methods

### Study design and subject enrolment

A well-designed systematic study was conducted. We first identified low expression of *NOS1* and promoter hypermethylation in CRC using RNA-Seq and HM450 data from the UCSC Xena database. *NOS1* hypermethylation was primarily confirmed by DNA methylation data generated using MassARRAY EpiTyper in 48 pairs of CRC and normal tissues in in-house Dataset 1. Furthermore, *NOS1* methylation status was assessed in 286 CRCs, 81 AAs, and 81 NAAs in our in-house dataset 2 to fully characterize the patterns of methylation changes in *NOS1* during CRC development. Additionally, 68 CRCs, 45 AAs, and 40 NAAs in in-house Dataset 3 were included to quantify the transcription levels of *NOS1*. The basic characteristics of the study patients are shown in Additional File 1 (Table S1). Finally, CRC patients with successfully measured DNA methylation data in in-house datasets 1 and 2 were pooled to evaluate the association of *NOS1* hypermethylation with CRC-specific survival. The enrolment of patients with CRC was based on data from the Shaoxing People’s Hospital between January 2015 and July 2018. Patients with colorectal adenomas (AA or NAA) were drawn from an ongoing population-based cohort since 1989 in Jiashan County, Zhejiang Province, China, which has been described in detail previously [23]. All patients were pathologically confirmed, excluding those with familial adenomatous polyposis (FAP), a previous history of CRC, and preoperative anticancer treatment. A histologically confirmed colorectal lesion (carcinoma or adenoma) sample and corresponding adjacent normal mucosa sample were obtained from each patient. Adjacent normal tissue samples were collected 5 cm from the primary neoplasm. Adenomas with at least one conventional adenoma ≥ 1 cm in diameter or advanced histology (tubulovillous/villous histological features or high-grade or severe dysplasia) were defined as AA, and adenomas < 1 cm in diameter without advanced histology were defined as NAA [24]. The pathological stages of CRC were classified into I, II, III, and IV according to the American Joint Committee on Cancer (AJCC) Cancer staging manual [25]. Our study was conducted according to the guidelines of the Declaration of Helsinki, and approved by the Ethics Committee at Zhejiang University School of Medicine (#2019–052, 2019 updated). Written informed consent was obtained from all individual participants included in the study.

### Public database analysis

We obtained RNA sequencing (RNA-Seq) and DNA methylation (HM450) data for 33 types of cancer and clinical data for CRC from the UCSC Xena database (http://xena.ucsc.edu/). A total of 11 CpG sites (cg20285745, cg04883903, cg03538436, cg04529785, cg04899175, cg20840426, cg18834729, cg02962597, cg21006686, cg21273407, cg18243853) located in the *NOS1* promoter were used. In addition, we downloaded RNA expression data (GSE44076, GSE21510, GSE18105, and GSE73360) from patients with CRC from the GEO database (https://www.ncbi.nlm.nih.gov/geo/).

### DNA isolation and bisulfite modification

Genomic DNA from fresh-frozen tissues was extracted using a DNA Tissue Kit (TianLong Biotech, Xi’an, China) and treated with sodium bisulphite using an EZ Methylation Gold Kit (Zymo Research, Irvine, CA, USA). All procedures were conducted according to standard protocols.

### Sequenom EpiTYPER assay

Quantitative DNA methylation analysis of the *NOS1* promoter was performed using MassARRAY EpiTyper (Sequenom, San Diego, CA, USA). Primers were designed using the online software EpiDesigner (https://epidesigner.com/, Additional file 1: Table S2). The target region contained 49 CpG sites, of which 43 were successfully detected. Close neighbouring CpG sites that could not be discriminated by the MassARRAY assay were analysed as CpG units. Therefore, the 43 CpG sites were combined into 29 CpG units for subsequent analyses. CpG units that yielded data in more than 80% of the samples, as well as samples with methylation calling in greater than 80% of CpG units, passed quality control. In in-house dataset 1, 28 CpG units were included in the analyses because of the low detection rate of CpG_9 (59.38%). In in-house dataset 2, 27 CpG units were included in the analyses as CpG_24 was further excluded due to the lower detection rate (75.89%) (Additional file 1: Figure S1). The beta values of these CpG units were between 0 and 1, and the average value of all successfully detected CpG units was calculated as the mean methylation level of *NOS1* promoter.

### RNA isolation, reverse transcription, and qRT‒PCR

Total RNA was extracted from the tissue samples using TRIzol reagent (Invitrogen, Carlsbad, CA, USA) according to the manufacturer’s instructions. Purified RNA was reverse-transcribed into cDNA using Moloney murine leukemia virus (M-MLV) reverse transcriptase (Takara, Otsu, Shiga, Japan). Amplification was performed using SYBR Premix Ex Taq (Takara, Otsu, Shiga, Japan) in a Light Cycler 480 (Roche Applied Science, Mannheim, Germany). The housekeeping gene, GAPDH, was used as an internal reference. The experiments were repeated in triplicate. The relative expression of *NOS1* was calculated using the 2^−ΔΔCT^. The specific primers used for qRT-PCR are listed in Additional File 1 (Table S2).

### Gene set enrichment analysis

GSEA determines whether a defined set of genes is significantly different between two biological states [26]. The c2.cp.kegg.v2023.1. The KEGG subset of curated gene sets (Hs. symbols. gmt) gene set database was used in this study. Gene expression data were analysed to identify differentially expressed genes (DEG) using the edgeR package. GSEA was employed to elucidate functional differences in the genes between *NOS1* low expression and *NOS1* high expression groups. The grouping was based on the proportion of ‘*NOS1* low’ to *‘NOS1* high’ within the studied CRC samples. FDR, normalized enrichment score (NES), and *p*-value were analysed to classify each phenotypic enrichment signalling pathway. To further dissect the functional implications of *NOS1*, functional annotation analysis was performed using Gene Ontology (GO) and Kyoto Encyclopedia of Genes and Genomes (KEGG) databases, which characterized the biological processes, molecular functions, cellular localizations, and signalling pathways in which *NOS1* is involved.

### Statistical analysis

The results are shown as the mean and standard deviation (SD) for continuous variables and as numbers and percentages for categorical variables. Paired Student’s t-test and Wilcoxon test were used to evaluate the differences in DNA methylation and expression levels between lesion and normal tissues. The relationship between DNA methylation and gene expression levels was tested using Spearman’s correlation. Analysis of variance (ANOVA) was used to perform multiple comparisons between groups. In the survival analysis, an optimal cutoff employing the Youden index based on the time-dependent ROC curve was used to dichotomize the study patients into high-risk and low-risk groups. Kaplan-Meier curves of disease-specific survival (DSS) were generated, and a log-rank test was performed to assess survival differences between the groups. The prognostic value was analysed using a multivariate Cox regression model adjusted for age, sex, and TNM stage. Hazard ratios (HRs) and 95% confidence intervals (95% CIs) were calculated.

Statistical analyses were performed using R, version 4.2.3. All tests were two-sided, and statistical significance was set at *p* < 0.05.

## Results

### Low expression and hypermethylation of NOS1 in human cancers by online database

**Figure 1.**
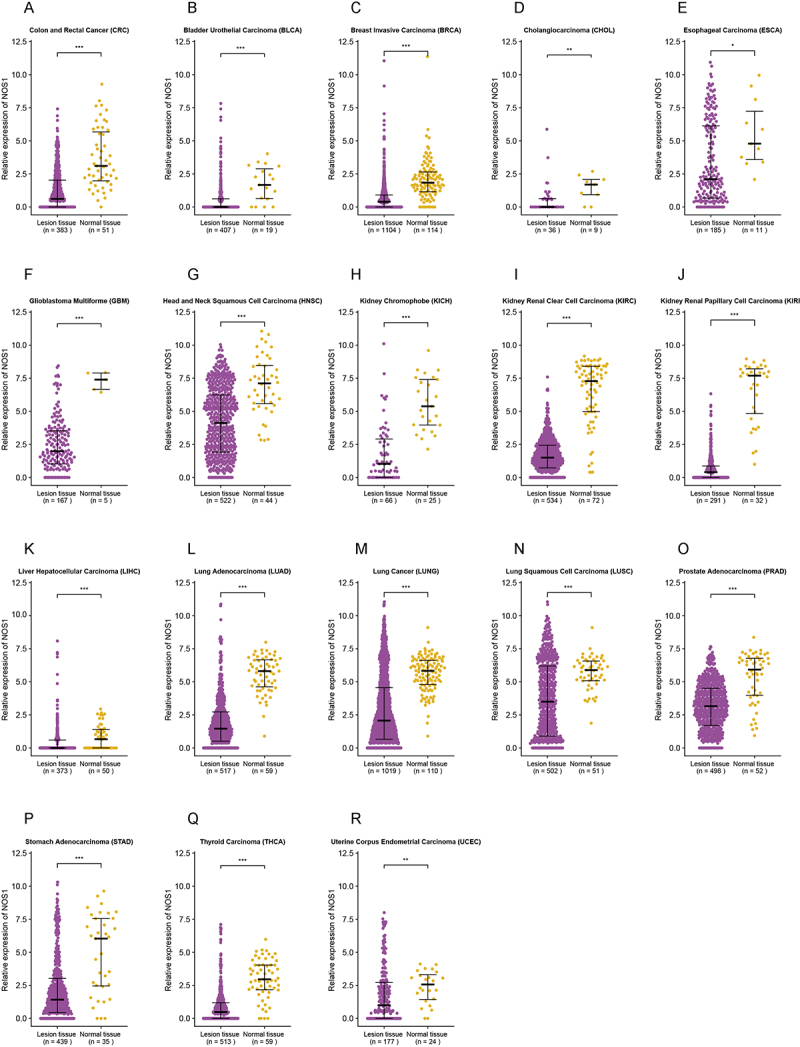
Differential *NOS1* expression in 18 cancers from the UCSC Xena database. Each dot represents the expression level in a tissue sample. A-R CRC, colorectal cancer; BLCA, bladder cancer; BRCA, breast cancer; CHOL, cholangiocarcinoma; esca, esophageal cancer; GBM, glioblastoma; HNSC, head and neck cancer; KICH, kidney chromophobe; KIRC, kidney clear cell carcinoma; KIRP, kidney papillary cell carcinoma; LIHC, liver cancer; LUAD, lung adenocarcinoma; lung, lung cancer; LUSC, lung squamous cell carcinoma; prad, prostate cancer; stad, stomach cancer; THCA, thyroid cancer; UCEC, endometrioid cancer. ***p < 0.001; **p < 0.01; *p < 0.05.

**Figure 2.**
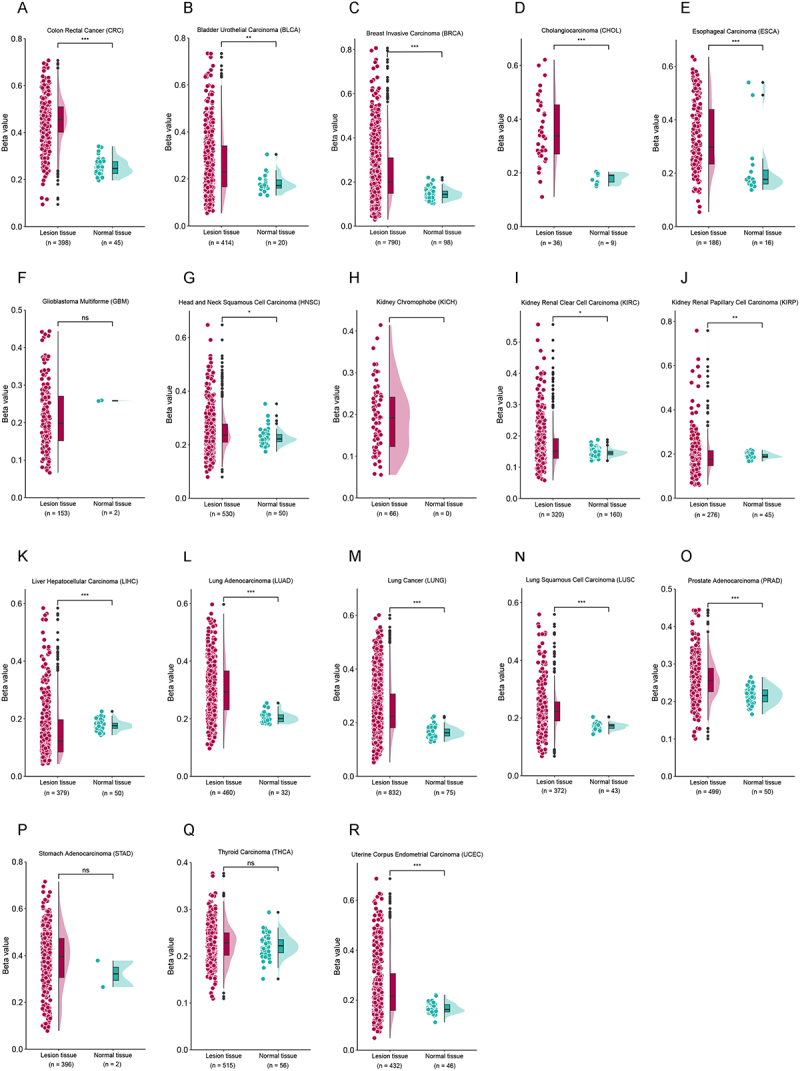
Analysis of *NOS1* methylation levels among 18 cancers with differential expression from UCSC Xena database. Each dot represents the methylation level for one tissue sample. A-R CRC, colorectal cancer; BLCA, bladder cancer; BRCA, breast cancer; CHOL, cholangiocarcinoma; esca, esophageal cancer; GBM, glioblastoma; HNSC, head and neck cancer; KICH, kidney chromophobe; KIRC, kidney clear cell carcinoma; KIRP, kidney papillary cell carcinoma; LIHC, liver cancer; LUAD, lung adenocarcinoma; lung, lung cancer; LUSC, lung squamous cell carcinoma; prad, prostate cancer; stad, stomach cancer; THCA, thyroid cancer; UCEC, endometrioid cancer. ***p < 0.001; **p < 0.01; *p < 0.05; ^ns^ *p*>0.05.

Differential expression analyses of *NOS1* were conducted between tumourous and normal tissues among 33 cancers from the UCSC Xena database. Compared to normal tissues, significantly lower *NOS1* expression was found in tumourous tissues of 18 cancers (*p* < 0.05) (Figure 1(A–R), Additional file 1: Figure S2A-O), of which 12 cancers showed significant hypermethylation in *NOS1*, with the greatest difference observed in CRC (Δβ = 0.19) (Figure 2(A–R)).

### Confirmation of NOS1 hypermethylation in in-house colorectal cancers

**Figure 3.**
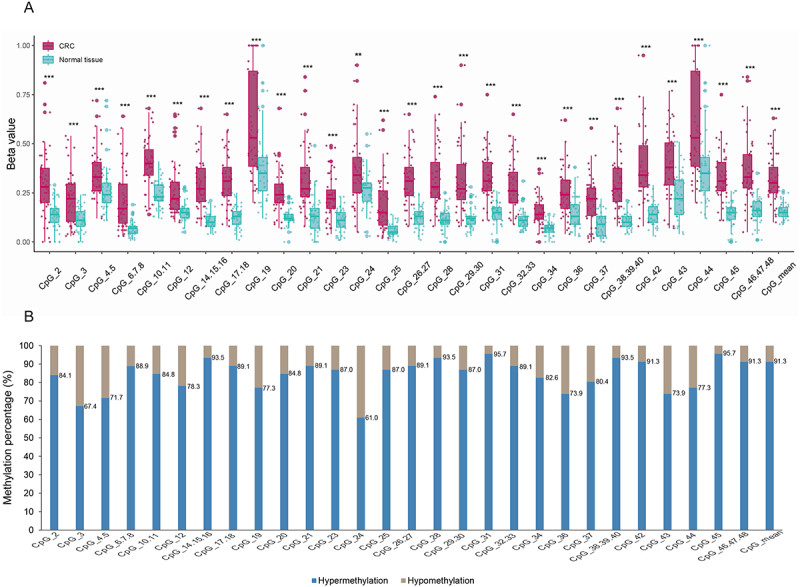
*NOS1* hypermethylation among CRC patients in in-house dataset 1. A methylation levels for individual CpG units and CpG_mean in 48 CRC patients. ***p < 0.001; **p < 0.01. B percentages of patients with *NOS1* hypermethylation for individual CpG units and CpG_mean. CRC, colorectal cancer.

The *NOS1* methylation status in 46 of the 48 pairs of colorectal tumourous and adjacent normal tissues was successfully measured. The mean and individual methylation levels of 28 CpG units in *NOS1* promoter were significantly higher in CRC tumourous tissues than in adjacent normal tissues (*p* < 0.01) (Figure 3A). Specifically, *NOS1* hypermethylation was found in 91.3% (42/46) of CRC patients (Figure 3B).

### Elucidation of NOS1 hypermethylation patterns at different stages of colorectal carcinogenesis

**Figure 4.**
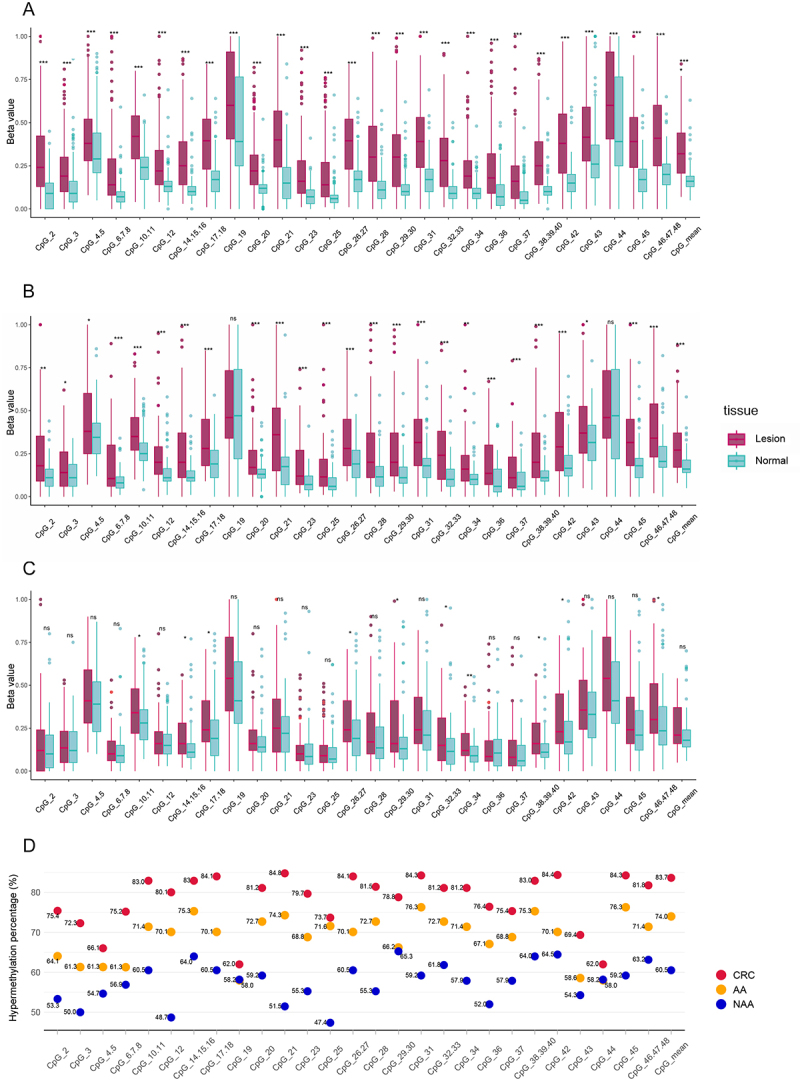
*NOS1* hypermethylation among patients at all stages of colorectal neoplasms in in-house dataset 2. A colorectal cancer (*n* = 286). B advanced adenoma (*n* = 81). C non-advanced adenoma (*n* = 81). Methylation levels for individual CpG units and CpG_mean in lesion tissues and paired normal tissues. ***p < 0.001; **p < 0.01; *p < 0.05; ^ns^ *p*>0.05. D percentages of patients with *NOS1* hypermethylation status.

To elucidate the pattern of *NOS1* methylation changes during colorectal carcinogenesis, the methylation status of *NOS1* was successfully detected in lesions and adjacent normal tissues of 286 CRCs, 81AAs, and 81 NAAs in in-house Dataset 2. The methylation levels of *NOS1* were consistently higher in CRC (Figure 4A) and AA (Figure 4B) tissues than in their corresponding normal tissues, but not in the comparison between NAA and their adjacent normal tissues (Figure 4C). Correspondingly, *NOS1* hypermethylation in lesion tissues occurred most frequently in CRC (83.7%, 231/276), intermediately in AA (74.0%, 57/77), and least frequently in NAA (60.5%, 46/76) (Figure 4D). Except for CpG_4.5, CpG_19, and CpG_44, significant hypermethylation was observed in most of the CpG units. Furthermore, aberrant hypermethylation changes increased from NAA to AA and CRC (*p*_trend_ < 0.001) (Additional file 1: Table S3).

### Affirmation of NOS1 expression status at different stages of colorectal cancer development

**Figure 5.**
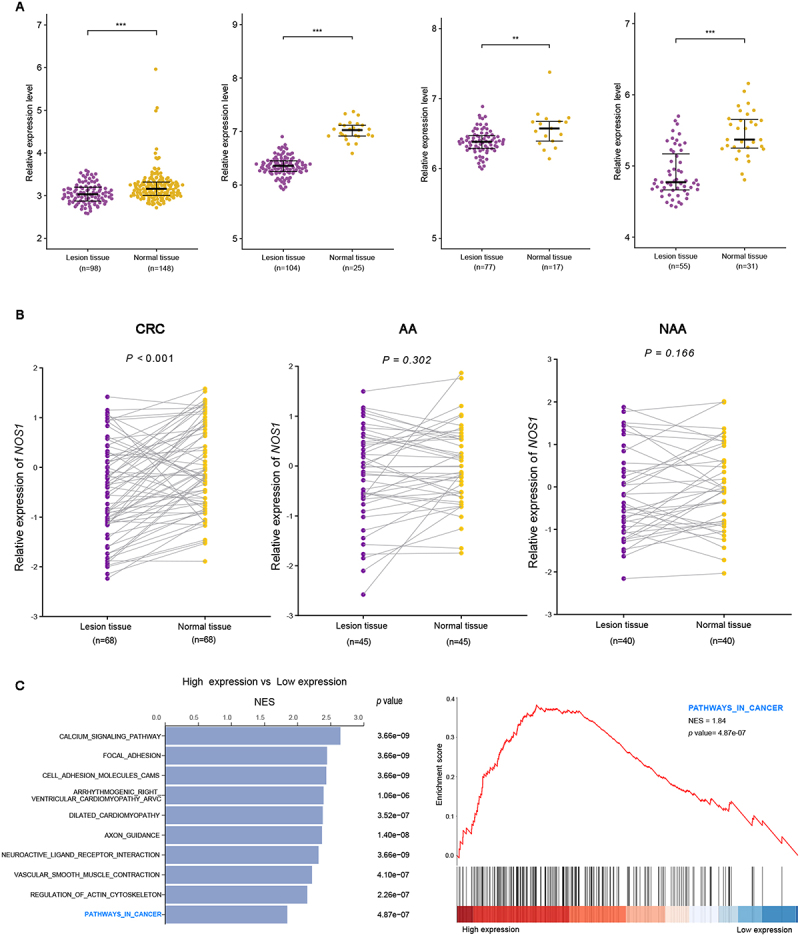
*NOS1* expression among patients at different stages of colorectal neoplasms in GEO database and in-house dataset 3 and gene set enrichment analysis (GSEA) using UCSC Xena database. A GSE44076, GSE21510, GSE18105, GSE73360 B colorectal cancer (*n* = 68), advanced adenoma (*n* = 45), non-advanced adenoma (*n* = 40). C GSEA of *NOS1* expression using RNA count data from CRC patients. NES, normalized enrichment score.

We further explored *NOS1* expression in CRC tissues using GEO and found that *NOS1* expression was significantly reduced in CRC tissues (Figure 5A). Furthermore, *NOS1* expression was detected in paired lesions and adjacent normal tissues from 68 CRCs, 45 AAs, and 40 NAAs in in-house Dataset 3. Significantly lower *NOS1* expression was found in CRCs (*p <* 0.001) (Figure 5B), but not in AAs (Figure 5B) or NAAs (Figure 5B). To further explore the potential mechanisms of *NOS1* gene in CRC, GSEA was conducted based on RNA count data from the UCSC Xena database. Pathway analyses showed that *NOS1* high expression was positively correlated with the ‘PATHWAYS_IN_CANCER’ signature (Figure 5C). Based on GO and KEGG annotations, we found that *NOS1* was involved in several biological pathways relevant to CRC, including nitric oxide production, hypoxia response, and cGMP-mediated signalling. *NOS1* also participated in arginine and nitrogen metabolic pathways that influence redox balance, vascular regulation, and tumour immune microenvironment (Additional file 1: Table S4, Table S5).

### Exploration of the prognostic significance of NOS1 in colorectal cancer patients

**Figure 6.**
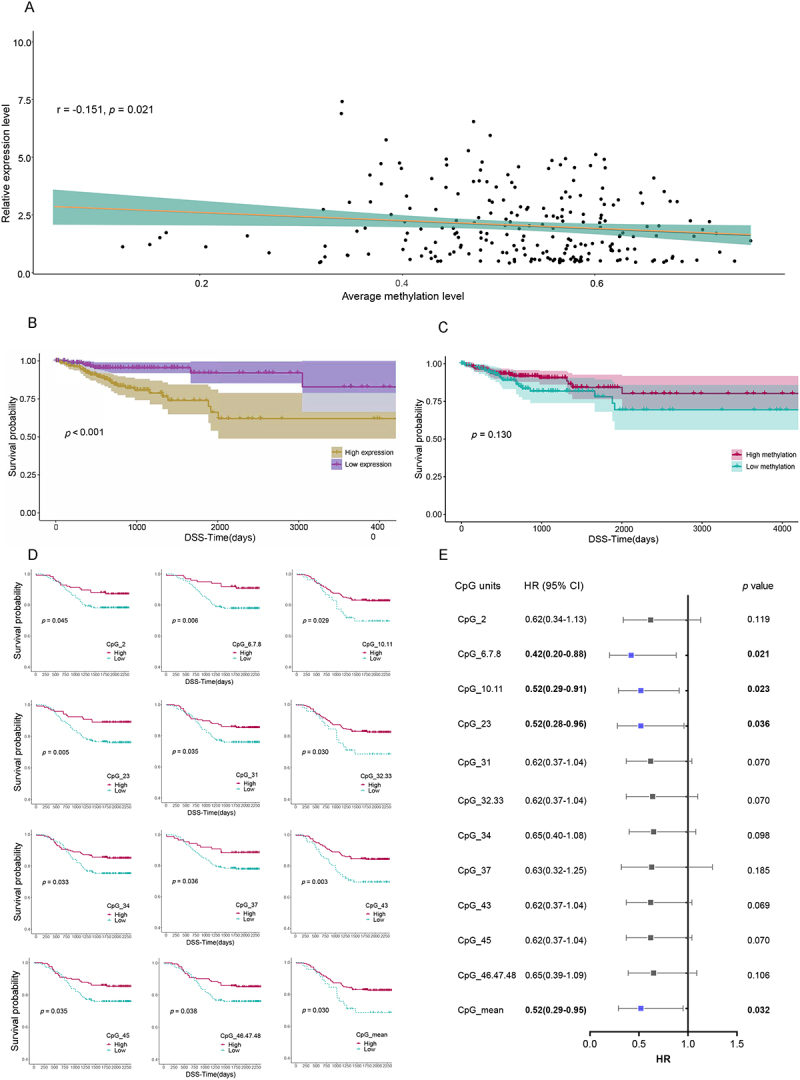
Survival analyses on CRC patients in UCSC Xena database and our in-house datasets. A the relationship between *NOS1* methylation and its mRNA expression in CRC patients (UCSC). B DSS in CRC patients stratified by expression levels (UCSC). C DSS in CRC patients stratified by methylation levels (UCSC). D DSS in CRC patients stratified by methylation levels (in-house dataset). E forest plot of hr (95% CI) by multivariate Cox regression analysis, adjusted for age, sex, and TNM stage (in-house dataset). CRC, colorectal cancer; DSS, disease-specific survival; hr, hazard ratio; CI, confidence interval.

Using the UCSC Xena database, we found that the *NOS1* expression was significantly negatively correlated with methylation levels in four of the 18 cancers, including CRC (*r* = −0.151, *p* = 0.021) (Figure 6A, Additional file 1: Table S6). Correspondingly, we further found that patients with low expression had significantly better survival than those with high expression, and those with high methylation had better survival than those with low methylation (Figure 6B-C).

In addition, we included 327 CRC patients with complete DNA methylation data from in-house datasets 1 and 2 for the survival analyses. There were 70 CRC-specific deaths, with a median follow-up time of 4.62 years. As shown in the Kaplan – Meier survival curves, 11 of the 27 CpG units were significantly associated with CRC survival. Patients with high *NOS1* methylation levels had better survival than those with low methylation levels (Figure 6D, Additional file 1: Figure S3). Multivariate Cox regression analysis revealed that *NOS1* methylation was an independent prognostic biomarker for patients with CRC (HR = 0.52, 95% CI: 0.29–0.95, *p* = 0.032) (Figure 6E).

## Discussion

The current understanding of *NOS1* methylation status during colorectal carcinogenesis is limited. In this study, using an online database, we first found *NOS1* low expression in 18 cancers, among which 12 were identified to be significantly hypermethylated, including CRC. Significant *NOS1* hypermethylation was confirmed in patients with CRC. In addition, significant *NOS1* hypermethylation was observed during colorectal carcinogenesis and the methylation level increased as the disease progressed from AA to CRC. However, *NOS1* lower expression was observed only in our in-house CRC tissue samples. Lastly, based on an online database and in-house datasets, we confirmed *NOS1* hypermethylation is a potential prognostic biomarker for patients with CRC.

DNA methylation, a crucial aspect of epigenetic regulation, has been shown to play an important role in CRC. Promoter hypermethylation of specific genes has been studied in CRC [27,28] and numerous studies have highlighted that DNA promoter hypermethylation can be involved in cancer development by inactivating tumour suppressor genes [29,30]. However, much remains unknown about the role of *NOS1* in the development of CRC, especially in terms of the association between its methylation, expression, and functionality. In this study, low expression and hypermethylation of *NOS1* were identified in 12 of 33 types of cancers, including CRC, and these findings were further validated in CRC patients from our in-house datasets. Although low expression of *NOS1* was only found in our in-house CRC patients but not in AA and NAA patients, a relationship between high methylation and low expression of *NOS1* was observed in CRC development. In addition, *NOS1* low expression and hypermethylation are associated with favourable prognosis in CRC patients. Specifically, *NOS1* hypermethylation was found to be an independent prognostic biomarker for CRC patients. Our findings are consistent with those of other studies [31,32], which have reported that methylation of genes, such as *MGMT* hypermethylation, is associated with a better prognosis in CRC patients. Moreover, high *NOS1* expression in CRC tissues is considered a risk factor in a CRC prognostic model [22]. Therefore, CRC patients with *NOS1* hypermethylation and corresponding low expression may be potential biomarkers for better CRC survival.

Our current study provides insights into the adenoma-to-cancer progression sequence of CRC. Epigenetic changes have been reported to occur during the transition from a normal state to precancerous lesions to a cancerous state. Nevertheless, most previous studies have mainly focused on bulk profiling of colorectal tumours, with limited attention given to premalignant lesions [33]. Colorectal adenomas are the most common precancerous lesions, and advanced adenomas are believed to have an increased potential for malignant transformation [34]. Studying these premalignant lesions could help to elucidate the biology of colorectal tumorigenesis. In our study, significantly different *NOS1* methylation levels were identified between the different stages of colorectal lesions. Hypermethylation of *NOS1* was found in 83.7%, 74.0%, and 60.5% of patients with CRCs, AAs, and NAAs, respectively, which supports the view that aberrant methylation patterns appear early during colorectal neoplastic progression [35]. Furthermore, *NOS1* hypermethylation was more common in the CpG units of AA than in NAA. This indicates that the biological mechanism underlying AA occurrence is much closer to the tumour stage than that of NAA and that accumulation of aberrant methylation is mostly identified at the advanced adenoma stage [32]. Taken together, *NOS1* hypermethylation has the potential to be a biomarker for early CRC detection. Our findings may help provide clues for future clinical practice, as methylation has been recognized as a stable epigenetic change [36].

Findings from previous studies have suggested the involvement of *NOS1* in CRC pathogenesis. For example, Wang et al. revealed that mitochondrial NOS1 inhibits apoptosis in colon cancer cells [37], and animal models of colitis showed impaired NOS1 expression [38]. Notably, NOS1, along with NOS2 and NOS3, constitute the nitric oxide synthase (NOS) family, releasing biologically active NO free radicals as a byproduct [39]. NO is one of the most important messengers capable of regulating diverse biological activities in cells and tissues [40]. Moreover, NO has been shown to have both tumoricidal and tumour-promoting effects depending on its timing, location, and concentration [41,42]. Previous studies have shown that all three isoforms may be involved in promoting or inhibiting the etiology of cancer by producing NO [19,43,44]. Methylation alterations can be pervasive, whereas the genes affected tend to be cancer-specific and involve multiple signaling pathways [45]. Research has found that high expression of *NOS2* increases DNA methyltransferase activity in intestinal metaplasia (IM) of precancerous lesions, thereby accelerating abnormal DNA methylation [46]. High expression of *NOS3* was observed associated with metastasis in gastric cancer and liver cancer [47]. Previous studies have found that NO can inhibit the activity of DNA methyltransferase 3B (DNMT3B) through nitrosylation modification. Interestingly, our analysis revealed distinct methylation and expression patterns of *NOS1* in CRC. This divergence likely reflects their different physiological roles and regulatory contexts. *NOS2* is often upregulated in inflammatory microenvironments and has been implicated in promoting tumour progression through NO-mediated DNA damage and angiogenesis [48]. *NOS3* is a key regulator of vascular homeostasis, and its overexpression may enhance tumour metastasis [47]. In contrast, *NOS1* is predominantly expressed in neuronal tissues and the enteric nervous system (ENS), and its silencing may be linked to ENS dysfunction or the loss of a tumour-suppressive NO signalling axis in colorectal epithelial cells [15,16]. This isoform-specific pattern underscores the unique role of *NOS1* methylation as a potential biomarker in CRC development and prognosis.

GSEA results showed that the high-*NOS1* group was enriched in cancer-related pathways, as well as several other pathways, and *NOS1* downregulation was related to a reduction in cancer-related pathways. Further detailed GO and KEGG pathway analyses further delineated the specific biological processes and signalling pathways associated with *NOS1* in CRC. Notably, *NOS1* was implicated in nitric oxide biosynthesis, the response to hypoxia, and cGMP-mediated signalling. *NOS1* also participated in arginine and nitrogen metabolic pathways that influence redox balance, vascular regulation, and tumour immune microenvironment. These pathways are intricately linked to colorectal carcinogenesis [49,50]. There is considerable molecular heterogeneity among colorectal tumours, resulting in different tumorigenesis pathways [9,38]. The enteric nervous system (ENS) is an understudied component of the tumour microenvironment, and previous research has highlighted its role in digestive system cancers [51,52]. However, its role in the development of CRC remains poorly understood [53]. Interestingly, genes associated with ENS, such as NDRG4 [54], were in the set of best-performing CRC-specific biomarkers. *NOS1* is also linked to ENS and shows a positive correlation with NDRG4 when analysed in an online database. Moreover, there was a significant correlation between methylation changes related to the nervous system and the early formation of colorectal adenomas [55]. Beyond establishing *NOS1* hypermethylation as a promising biomarker for risk estimation during colorectal carcinogenesis and prognosis, our functional enrichment analyses illuminate its potential roles in critical cancer relevant pathways – from NO signalling and hypoxia adaptation to immunometabolic regulation. Taken together, we infer a potential association between the nervous system, methylation changes, and the development of CRC.

A potential counterintuitive observation in our study is that *NOS1* hypermethylation and reduced expression were associated with improved survival in CRC patients. Although *NOS1* hypermethylation occurs during CRC progression, this favourable prognostic association can be explained from several biological perspectives. First, *NOS1*-derived NO exhibits context-dependent functions in cancer. While low levels of NO may support cell survival, excessive NO production can trigger oxidative stress, DNA damage, and chronic inflammation [41,42], all of which promote tumour progression and therapy resistance. Therefore, epigenetic silencing of *NOS1* may reduce excessive NO generation and restrict tumour aggressiveness. Second, *NOS1* hypermethylation may serve as a surrogate marker for less malignant tumour phenotypes, such as better differentiation or earlier tumour stage, rather than directly determining prognosis. Third, the function of *NOS1* in CRC is distinct from that of other NOS isoforms and remains incompletely characterized. Our results highlight the complex and context-specific roles of *NOS1* in colorectal tumorigenesis and support the value of *NOS1* methylation as a prognostic biomarker.

Notably, no previous study has investigated *NOS1* hypermethylation in CRC development and prognosis. Nevertheless, our study has some limitations. First, although our study suggests that *NOS1* hypermethylation may play an important role in the progression of adenoma to carcinoma, further functional studies are needed to elucidate its mechanism in colorectal carcinogenesis. Second, validation with larger samples, diverse study populations, and biological sample types (cells, etc.) is needed to verify the consistency of the current results. Finally, the relationships between *NOS1* methylation levels and well-known prognostic markers, such as CpG island methylation phenotype (CIMP), BRAF, and RAS mutations, could not be explored in our study, as these data were unavailable for our in-house patients or the UCSC Xena database. Longer clinical surveillance and further adjustment of other relevant factors are needed to provide evidence for the clinical value of this epigenetic biomarker.

## Conclusion

In summary, based on the systematic elucidation of low expression and hypermethylation of *NOS1* in multiple cancers using online databases, its hypermethylation was further confirmed in colorectal cancer and advanced adenoma using our in-house database, with steadily increasing levels from advanced adenoma to cancer. Moreover, *NOS1* hypermethylation was significantly associated with better survival in colorectal patients. Thus, it may be a promising biomarker for risk estimation during colorectal carcinogenesis and prognosis.

## Data Availability

RNA-Seq, HM450 methylation, and clinical data for 33 cancers used in the study can be accessed at the UCSC Xena database (https://xena.ucsc.edu/) and the GEO database (https://www.ncbi.nlm.nih.gov/geo/). The data that support the findings of this study are openly available in figshare at DOI: 10.6084/m9.figshare. 30,879,791, reference number 30879791.

## Abbreviation list

AA: advanced adenoma
ANOVA: analysis of variance
CpG: cytosine-guanine dinucleotide
CRC: colorectal cancer
DSS: disease-specific survival
FAP: familial adenomatous polyposis
FDR: false discovery rate
GSEA: gene set enrichment analysis
HM450: Illumina HumanMethylation450 Bead Chip
M-MLV: Moloney Murine Leukemia Virus
NAA: non-advanced adenoma
NES: normalized enrichment score
*NOS1*: nitric oxide synthase 1
qRT-PCR: quantitative reverse transcription-polymerase chain reaction
RNA-Seq: RNA sequencing
SD: standard deviation.
